# Looking Like a Million Dollars: Does Attractiveness Priming Increase Altruistic Behavior in Experimental Games?

**DOI:** 10.3389/fpsyg.2021.658466

**Published:** 2021-07-20

**Authors:** Julie Novakova, Kamila Machová, Katerina Sýkorová, Vojtěch Zíka, Jaroslav Flegr

**Affiliations:** ^1^Laboratory of Evolutionary Biology, Department of Philosophy and History of Science, Faculty of Science, Charles University, Prague, Czechia; ^2^Center for Behavioral Experiments (CEBEX), Prague, Czechia; ^3^Applied Neuroscience and Neuroimaging, National Institute of Mental Health, Klecany, Czechia

**Keywords:** altruism, attractiveness, costly signaling, experimental games, reputation, sexual selection

## Abstract

The emergence of altruistic behavior constitutes one of the most widely studied problems in evolutionary biology and behavioral science. Multiple explanations have been proposed, most importantly including kin selection, reciprocity, and costly signaling in sexual selection. In order to test the latter, this study investigated whether people behave more altruistically when primed by photographs of attractive faces and whether more or less altruistic people differ in the number of sexual and romantic partners. Participants in the general population (*N* = 158, 84 F, 74 M) first rated the attractiveness of photographs of 20 faces of the opposite (sexually preferred) sex and then played the Dictator and Ultimatum Games (DG and UG). The photograph rating acted as priming; half the participants received photographs of people rated as more attractive than average in an earlier study, and the other half received photographs previously rated as less attractive. The attractiveness-primed participants, especially men, were expected to behave more altruistically—signaling that they are desirable, resource-possessing partners. We also expected altruists to self-report more sexual and romantic partners. The observed difference between altruistic behaviors in the attractiveness- and unattractiveness-primed groups occurred in UG offers, however, in the opposite than expected direction in women. The number of sexual partners was positively correlated to minimum acceptable offers (MAOs) in the UG, in line with expectations based on the theory of costly signaling.

## Introduction

“To ignore the questions of survival value and evolution… makes it impossible to arrive at an understanding of behavioral problem.”

– Niko Tinbergen (1963).

Evolutionary explanations for altruistic behavior, fairness, and cooperation continue to garner debate even after decades of research studies. Theories ranging from mutualism, kin selection, interdemic selection, reciprocal altruism (direct or indirect, based on individual reputation), and “greenbeard” altruism to costly signalization and sexual selection have been proposed (Fehr and Fischbacher, 2003; West et al., 2007; Clutton-Brock, 2009). Most are not mutually exclusive and could have contributed to the evolution of altruistic behavior to some extent. Cooperation among humans may also have been shaped by factors that contributed less in other primate species, such as cooperative breeding and cultural evolution (Silk and House, 2016).

Explanations of the adaptive value of altruism and its evolutionary origins do not undermine the psychological mechanisms that produce altruism, because function and mechanism are each different levels of question (Tinbergen, 1963). That altruism may be of adaptive benefit to the altruist and, thus, ultimately “selfish,” does not diminish the psychological motivations for selflessly helping others: experiencing the oft-cited “warm glow,” emphatic concern, adherence to social norms, or displaying an intrinsic helping preference. Those are merely different level facets of the phenomenon that should not be confused (Clavien and Chapuisat, 2013; Kurzban et al., 2015). In the same vein, we should not “hijack” the mechanistic terminology of motivation when deriving evolutionary explanations (De Waal, 2008).

Altruism as a costly signal (driven by sexual selection; e.g., Zahavi, 1995) represents one of the major evolutionary theories of altruism and has received a lot of attention among evolutionary biologists, psychologists, and behavioral scientists studying humans. It is supported by experimental and theoretical data, including models of cooperation as an honest costly signal (e.g., Gintis et al., 2001). It may, however, be difficult to discern the various selection pressures contributing to altruistic behavior. Roberts (1998) suggested a basic criterion for distinguishing reciprocity from competitive altruism (i.e., sexual selection, along with general reputation and alliance building): If the altruistic act is returned later in a similar manner, reciprocity can be invoked (e.g., in allogrooming impalas and allofeeding vampire bats). If the act is not returned, competitive altruism may apply (possibly in primate allogrooming, bird allopreening, or group cooperation). Moreover, Shultziner and Dattner (2016) reasoned that the criteria for indirect reciprocity, an extension of the original reciprocal altruism of Trivers (1971), have become so loose that they make the distinction practically untestable. It blends into competitive altruism through reputation concerns, which are considered by both theories. In human, we can rarely observe a clear reciprocal “transaction,” even across longer timescales, and one-shot altruism is commonplace. This suggests that either altruism used to be based on reciprocity in people earlier but is now a maladaptation of the “paleolithic mind” (Trivers in Buchanan, 2005; Phillips, 2015) or that competitive altruism may be a more important driving force for altruism in humans.

Mate choice based on sexual preference for altruists is supported by a number of studies. In the presence of observers (or even observer cues such as a printed image of human eyes), people are more inclined toward generosity (Haley and Fessler, 2005; Bateson et al., 2006; Burnham and Hare, 2007; Bourrat et al., 2011; Ernest-Jones et al., 2011; Nettle et al., 2013; although the effect may be limited to short cue exposure: Sparks and Barclay, 2013), and they donate more under non-anonymous settings (Burnham, 2003). Competitive altruism, in general, seems common among humans (Van Vugt et al., 2007). These findings may be attributed to general social selection and reputation seeking (which includes sexual selection as the reputation of an altruist would be advantageous in mate choice).

The effect is even more pronounced in the presence of people of sexually desired sex of an individual, especially in men. Men are more likely to give money to beggars in the company of a woman, particularly in the early stages of a relationship (Latane, 1970), and also to give money to female beggars (Goldberg, 1995). Laboratory results indicate the same. Altruistic behavior is often measured through the Dictator Game (DG) (Forsythe et al., 1994) and the Ultimatum Game (UG) (Güth et al., 1982), both widely used in behavioral sciences. Both games have two players, who in typical laboratory settings cannot see each other or interact directly. In the DG, the first player is given an incentive (usually monetary) by the experimenter and can share it with the second player, who has no power over the outcome of the game. In the UG, the second player can decide whether to accept or reject the proposed offer. In the former, the second player receives the offered portion of the pie and the first player keeps the rest; in the latter, neither receives anything. Modified versions of both games have also been utilized in various animal cooperation studies (e.g., Jensen et al., 2007; Rutte and Taborsky, 2007, 2008; Kaiser et al., 2012; Proctor et al., 2013). DG giving is more clearly a sign of altruism, while the UG involves an element of strategic reasoning, making an offer that is unlikely to be rejected. Humans do not act as “pure gain-maximizers” without other motives, so not just any positive offer gets accepted. In most societies, an even split is the “surest bet,” though there is great variance both within and across societies (Henrich et al., 2006), and offers and rejection rates can be modulated by the stakes (Novakova and Flegr, 2013).

Both games have been used in previous studies that focused on altruistic behavior and physical attractiveness. Men tend to be more generous to the players of the opposite sex in the experimental games (Farrelly et al., 2007; Bhogal et al., 2016) or in the presence of third-party female observers (Iredale et al., 2008). Competitive altruism among men can also be observed in tasks that require some degree of self-sacrifice in order to obtain a reward for co-players (McAndrew and Perilloux, 2012). Even though the effect is more pronounced in men, it is not limited to them. Both sexes cooperated more with more attractive people of the opposite sex in several experimental games (Farrelly et al., 2007; Bhogal et al., 2016).

This pattern seems to occur across human societies. Foraging sex differences among hunter-gatherers may be caused by signaling benefits: Relatively inefficient, but difficult foraging strategies may persist chiefly among men due to costly signaling (Bird et al., 2001). Tognetti et al. (2012) studied cooperation in the Public Goods Game (PGG), played for rice and altruistic giving (*via* a donation test) in the presence of observers of either the same or opposite sex in rural Senegal. Men contributed more in the PGG when observed by either sex, but their contributions in the game gradually increased if observed by young women. Women contributed more in the presence of other women, but their contributions gradually increased when observed by men, although they did not exceed the overall altruistic behavior shown in the presence of other women. In the donation test, both sexes were more likely to donate in the presence of female observers. The existence and extent of the donations did not influence the perception of observers on the attractiveness of players. The results suggest that sexual selection may be driving the altruistic and cooperative behavior of men, whereas other types of social selection play a greater role in the altruistic behavior of women.

However, Saad and Gill (2001) did not detect any effect of the physical attractiveness of recipients on UG offers but only detected that men were more generous toward women. Similarly, Bhogal et al. (2017) found no relationship between the attractiveness of people and the level of altruistic behavior of others directly toward them in a face-to-face DG/UG. But their games were played for chocolate coins, and food, unlike money, acts as an immediate reinforcer in humans and other animals (Mazur, 1994; Stephens et al., 2006; Epstein et al., 2010), which might account for the null results. Finally, in terms of non-financial altruism, Schwarz and Baßfeld (2019) and Bhogal et al. (2019) found a positive effect of attractiveness in both sexes on willingness to help a stranger on a social network and in imaginary credit sharing, respectively.

There is also evidence that altruistic people are perceived as more attractive mates, especially in female choice. Phillips et al. (2008) found mate preference for altruism in both sexes, more strongly in women. Their twin study (Phillips et al., 2010) suggests there may be a genetic component in preference for altruism. Barclay (2010) presented volunteers with descriptions of people with varying traits, including altruism, and found that more altruistic people were considered to be more desired partners by both sexes. Arnocky et al. (2017) showed that more altruistic people (with this trait both self-reported and measured in a modified DG) had more self-reported sexual partners, suggesting the role of sexual selection in human altruistic behavior.

We aimed to investigate the effect of attractiveness in both ways, i.e., expecting subjects (especially men) to behave more altruistically in the DG/UG (both used in previous studies) after viewing photographs of attractive people of the opposite sex and expecting altruists to have more self-reported sexual and romantic partners. As potential confounders, we also measured the Big Five personality traits, where especially agreeableness was previously associated with altruism (Ashton et al., 1998). In addition, we investigated the effect of the self-perceived attractiveness of participants on their behavior in the experimental games. In distinguishing these effects in a single study, we are filling an important gap in the existing literature studies. Based on the theory of costly selection, attractiveness-primed people and people with more partners and self-perceived as more attractive should be more altruistic in giving and offering but also more demanding from others (= less willing to accept low UG offers) in order to keep or increase their relative social status.

## Materials and Methods

### Subjects and Course of the Experiment

The data were collected at the Charles University in Prague in June and July 2018/2019. Participants were invited *via* online recruitment on Facebook, with a maximum of 18 participants invited for each session, to take part in several tasks for a set of studies. Each session consisted of either men or women only in order to eliminate stochastic noise (the presence/absence of attractive opposite-sex individuals among the participants of an experimental session). Only self-reported heterosexuals were recruited for the study in order to clearly discern relationships between the measured variables on a sample of the target size. In total, 158 people participated in the experiments (74 men: mean age = 29, median of age = 29; 84 women: mean age = 31, median of age = 28).

Participants were greeted at the reception and led to the computer laboratory with seats separated by cardboard screens so that the participants could not see each other or otherwise interact during the experiment. They read and signed their informed consent and were able to ask questions about it before the commencement of the experiments. At the beginning of the computer-based survey, participants viewed 20 neutral-expression frontal portrait color photographs of people of the opposite sex. The priming part of the session was always overseen by an experimenter of the same sex as the participants.

The participants were asked to rate the attractiveness of the faces on an 8-point Likert scale [same as in the source study, Flegr et al. (2019), employed in order to avoid the “middle” response]. One half was randomly allocated photographs rated as less attractive on the same Likert scale by an independent sample in a previous questionnaire (Machová, 2018), and the other half was shown the 20 faces rated as more attractive. The unattractive sample of male photographs was originally rated 2.73 on average and the attractive was rated 3.97 (*t* = 7.98, df = 37.67, *p* < 0.0001) on an 8-point scale. The unattractive sample of female photographs had an average rating of 3.01 and the attractive was rated 5.21 (*t* = 9.64, df = 33.05, *p* < 0.0001) on the same scale. Differences of the priming sets in other characteristics are shown in Supplementary Table 1. The photographs used in the study of Machová (2018) and rated there for attractiveness belonged to materials from Flegr et al. (2019): randomly selected freely available photographs on the internet, where the selection criteria were estimated with ages 18–70, frontal view, neutral expression, resolution of at least 1,165 ×1,476 pixels, background, and hair possibly covered with a gray mask in postproduction.

Afterward, the subjects played first the DG and then the UG, each for 400 tokens (=40 CZK, approx. 2 USD), with no intermission. They were presented with the rules and truthfully told that they would be randomly matched with another participant in the room, and their earnings at the end of the experiment would reflect the outcome of both games. Each participant was asked how much they wanted to give and offer the other player, what is their minimum acceptable offer (MAO) in the UG, and also whether they would hypothetically accept or reject an offer of 120 tokens (30% of the pie). The survey contained control questions to assess whether the participants understood the rules of the games before they played and also a question about previous knowledge of the games.

The participants subsequently filled in a questionnaire asking about the number of sexual and romantic partners during the past year and their life so far. Three questions were aimed at self-perceived attractiveness of an individual in terms of both physical appearance (of the face and body of an individual) and personality on a 5-point scale. Big Five personality factors (agreeableness, extraversion, openness, neuroticism, and conscientiousness) were also measured *via* Ten-Item Personality Inventory (TIPI) (Gosling et al., 2003), as previous research studies found a connection for each of them with some aspects of altruism and cooperation, and they might modulate the responses of the participants (Clark et al., 2014; Oda et al., 2014).

Finally, in order to avoid the possible confounding effects of the status of an individual, the participants were also asked about their economic and social–family life satisfaction. Other data were collected for related studies on conditions of Triversian reciprocity and hormonal effects, respectively: health, temporal discounting, cognitive reflection, intelligence, memory, risk-taking propensity, facial photograph, 2D:4D, and salivary cortisol and testosterone levels. At the end of the testing, subjects were informed about the goals of the experiment and were paid their reward.

### Statistical Analysis

The analysis was conducted in R (R 3.6.1 for 64-bit Linux; R Core Team, 2013). Responses were checked for their validity (completion of the survey and answering control questions about understanding the principles of the games correctly), and the aggregate variables were computed (self-perceived attractiveness as added points from the perceived body, face, and personality attractiveness of an individual; personality traits from TIPI questions). Descriptive statistics were calculated first.

Afterward, a linear regression model was built for each of the studied dependent variables: allocation to another player in the DG, offer made to another player in the UG, the MAO in the UG, and reaction to the hypothetical offer of 120 tokens (30% of the pie) in the UG. In each starting model, the following predictors were used: participant sex, age, previous knowledge of the games, priming (and its interaction with sex), Big Five personality traits (and their interactions with sex), number of romantic partners in the previous year (and its interaction with sex), number of sexual partners in the previous year (and its interaction with sex), number of romantic partners in total (and its interaction with sex and age), number of sexual partners in total (and its interaction with sex and age), and self-perceived attractiveness of an individual (and its interaction with sex and age). Backward elimination was then applied to reach the best-fitting model.

With alpha set to 0.05, *N* = 158, 14 independent variables, and expected small effect size [estimated 0.14 for the purposes of the power analysis (PA)], the power of the test was 0.91, which was calculated using the R package “pwr” (Champely et al., 2018).

Finally, values of DG allocations and UG offers that are statistical outliers were kept in the analyses, because while DG and UG responses tend to have a strong central tendency to the mode, they also exhibit great range within and across different samples (Henrich et al., 2006), and outlier exclusion in this case would potentially cause a great distortion from reality.

The dataset used in our analyses has been published on FigShare (10.6084/m9.figshare.12067407).

## Results

### Descriptive Statistics

All 158 responses were valid, meaning that the participants finished the experiment and correctly answered the control questions. The mean DG allocation was 154.4 out of 400 tokens (~38.6%), median was 200 (50%), and SD was 76.2. The mean UG offer was 202 tokens (50.5%), median was 200 (50%), and SD was 51.8. One-sided Pearson's correlation test (expecting a positive correlation) showed both offers to be weakly correlated (*r* = 0.152, *t* = 1.924, df = 156, *p* = 0.028).

Mean MAO in the UG was 140.4 tokens (35.1%), median was 150 (37.5%), and SD was 69.6. Gender differences in allocations, offers, and MAOs were observed, but none were statistically significant (see Table 1). Finally, 56% (54% of women and 58% of men) of participants would accept a hypothetical offer of 120 tokens, while the rest would reject it. Previous knowledge of the games had no significant effect on the responses of the participants.

**Table 1:** Gender differences in DG and UG responses.

	**All participants**		**Women**		**Men**	
	**Mean**	**Median**	**Mean**	**Median**	**Mean**	**Median**
DG allocation	154.4 (38.6%)	200 (50%)	165 (41.25%)	200 (50%)	142.42 (35.61%)	200 (50%)
Statistical difference between sexes	–		*t* = 1.830, *df* = 125.6, *p* = 0.070			
UG offer	202 (50.5%)	200 (50%)	197.4 (49.35%)	200 (50%)	207.9 (51.98%)	200 (50%)
Statistical difference between sexes	–		*t* = −1.284, *df* = 155.9, *p* = 0.201			
UG MAO	140.4 (35.1%)	150 (37.5%)	148.2 (37.05%)	160.5 (40.13%)	131.5 (32.88%)	134 (33.5%)
Statistical difference between sexes	–		*t* = 1.509, *df* = 154.3, *p* = 0.133			

*The numbers in parentheses show the fraction of the maximum possible sum. The basic difference of response between sexes was measured by two-sampled t-tests*.

### Confirmatory Statistics

#### Dictator Game Allocations

The only significant predictor of DG offer that remained after performing backward elimination on the starting full linear regression model was the trait agreeableness (*t* = 2.269, SE = 5.291, *p* = 0.025): the higher an individual scored in agreeableness, the more they allocated to others in the DG.

#### Ultimatum Game Offers

**Figure 1:**
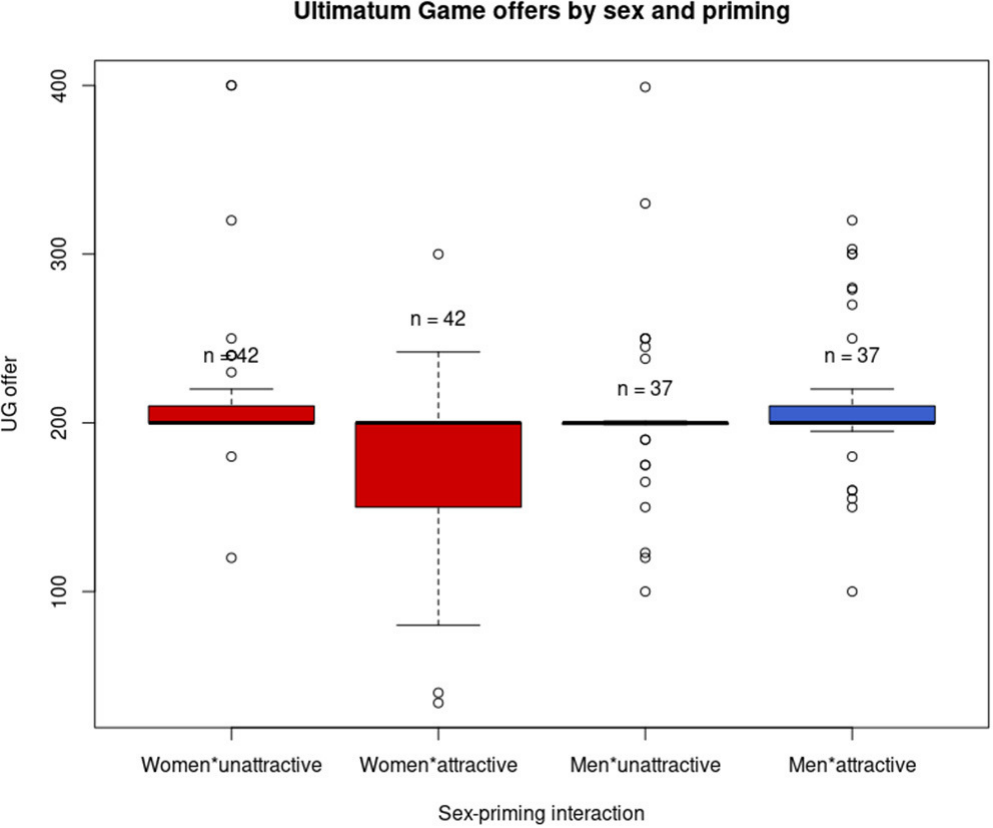
Ultimatum Game (UG) offers by participant sex (women shown in red and men in blue) and priming. Priming had a negative effect on UG offers in the overall sample and women. The difference in (un)attractiveness-primed women is visible in the two left boxplots. In men, no substantial difference was observed. The boxes show the interquartile range (IQR), and the bold line denotes the median. Whiskers can indicate either x 1.5 IQR from median or minimum and maximum in the absence of outliers (shown as circles).

UG offers were most strongly predicted by priming (*t* = −2.940, SE = 11.098, *p* = 0.004), followed by its interaction with sex (*t* = 2.862, SE = 16.184, *p* = 0.005), extraversion–sex interaction (*t* = −2.802, SE = 4.559, *p* = 0.006), neuroticism–sex interaction (*t* = 2.284, SE = 4.580, *p* = 0.024), trends with extraversion (*t* = 1.955, SE = 3.369, *p* = 0.052), and neuroticism (*t* = −1.807, SE = 3.584, *p* = 0.073) alone. Priming by attractiveness, therefore, led to lower UG offers overall and in women but increased offers by male participants (see Figure 1). Extraversion predicted higher offers in women but lower offers in men. An opposite effect was observed for neuroticism.

When the analysis was subsequently performed for each of the sexes separately, the significant negative effect of attractiveness priming was retained in women (*t* = −2.795, SE = 11.346, *p* = 0.007), while the effects of extraversion (*p* = 0.060) and neuroticism (*p* = 0.103) were not significant in this limited sample. None of the effects were significant in a subsample of men alone (priming *p* = 0.403, extraversion *p* = 0.143, and neuroticism *p* = 0.407).

#### Ultimatum Game Responses

**Figure 2:**
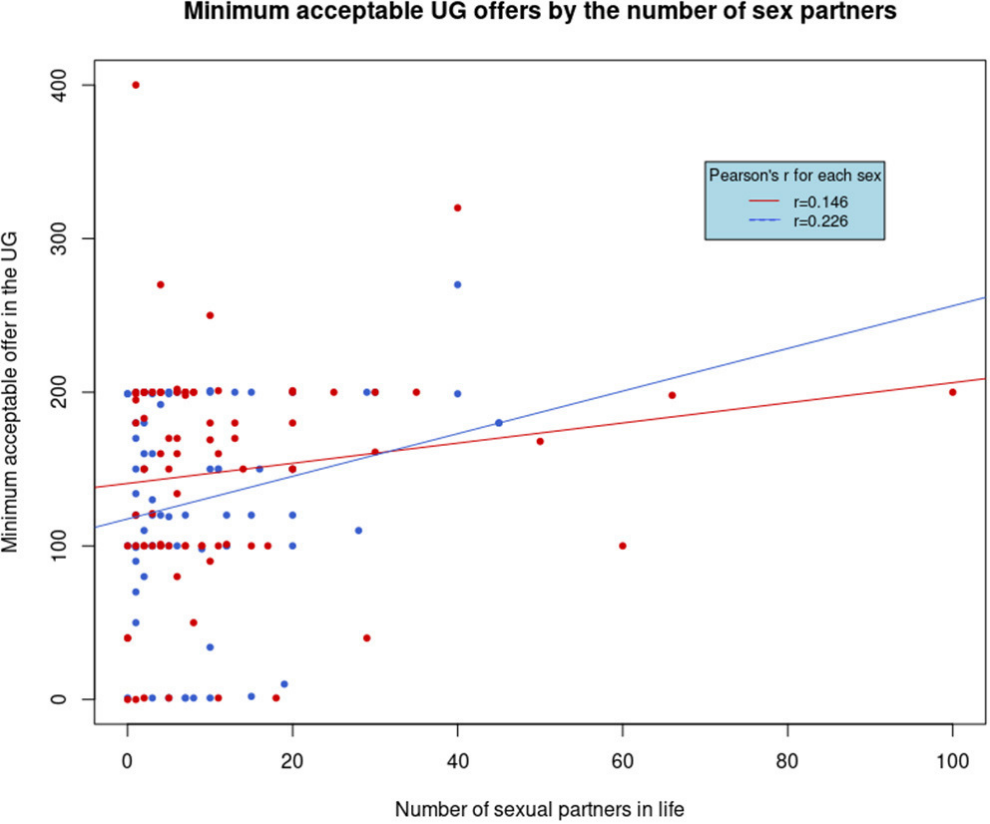
Minimum acceptable offer (MAO) in the UG by the number of sexual partners in life. Women are shown in red and men in blue. The regression line for each sex is displayed, and the rectangle shows Pearson's *r* for each sex.

The minimum acceptable UG offer was significantly (and positively) predicted only by the number of lifetime sexual partners (*t* = 2.269, SE = 0.399, *p* = 0.025; shown graphically in Figure 2), with no significant interactions or trends, and no predictors proved significant for the rejection or acceptance of the hypothetical offer of 120 tokens.

## Discussion and Conclusions

The behavior of participants in this study in both experimental games was roughly comparable to previous studies, where the average DG allocation is typically 25–30% of the pie, and the modal and median offers in the UG tend to be a fair split, with the average offer slightly above 40%. Offers of 20% or lower are more often rejected than those which are not, and offers of 30% also tend to be frequently rejected (e.g., Camerer, 2003; Engel, 2011; Tisserand et al., 2015). Our subject pool seems moderately more generous than the average, but well within the norm, and roughly conformed to previous findings in the same cultural setting (Lindová et al., 2010; Novakova and Flegr, 2013; Veselý, 2014).

We found no effect of attractiveness priming on DG allocations or responses to UG offers, but it had the expected effect on UG offers made by men and the opposite effect on offers made by women. In addition, we found that subjects of both sexes who were more demanding and who sought fairness in the UG had more self-reported sexual partners. This is in line with the theory of costly signaling. An UG offer rejection is a costly act that is commonly interpreted as altruistic punishment; however, we suggest that its role in establishing social rank and reputation is more probable.

The study finding that attractiveness priming positively influenced offers by men in the experimental situation of the UG is in line with the majority of studies so far (Farrelly et al., 2007; Bhogal et al., 2016, 2019; Schwarz and Baßfeld, 2019), whereas the observed opposite effect in women stands in contrast to them. While some previous research studies found no connection between attractiveness and altruistic behavior (Saad and Gill, 2001; Bhogal et al., 2017), this is to our knowledge the first occurrence of the opposite relationship in either sex. When we divided the sample by sex, the negative effect of priming remained significant in women (*n* = 84), while the positive effect in the smaller sample of men (*n* = 74) did not retain significance.

The curious negative effect of attractiveness on UG offers by women calls for explanation, and we can offer several speculations of such. It is worth pointing out that no effect manifested in the DG, which represents a clearer case of altruism, and its existence in the more complex UG may, therefore, be linked to the strategic element of the experimental situation.

While the participants were specifically primed for attractiveness by rating this trait of the faces on photographs, we cannot exclude the possibility that some other factors of the shown images may have influenced the decision-making of the participants. The original study of Machová (2018) included data not only for attractiveness but also for a number of other characteristics rated by volunteers online. The attractiveness of male faces rated by women correlated positively with most traits or descriptions (nice, an ideal partner for a one-night stand, an ideal life partner, sexy, charismatic, intelligent, fun, rich, generous, altruistic, trustworthy, healthy, mature, good skin quality, and symmetrical), weakly but positively with perceived faithfulness and maturity, and negatively with perceived masculinity and dominance. The attractiveness of female faces rated by men correlated positively with all traits but dominance and faithfulness. We can speculate that the halo effect of beauty (e.g., Zebrowitz and Franklin, 2014), i.e., the positive correlation of attractiveness with the majority of desirable traits and descriptions, could have enhanced the perception of another characteristic in our subjects, although attractiveness was made salient by the rating. Supplementary Table 1 summarizes the differences in various characteristics by the attractive/unattractive sample. Dominance, however, which may have seemed a likely potential cause of the results, did not significantly differ in either men or women. It is possible, though, that another trait or emotion could have been attributed to the faces, since neutral-expression faces are often ascribed emotional states by observers (Hester, 2019). On the contrary, it does not explain the negative effect exhibiting in female participants only.

We can also theorize that we observed no significant effect of attractiveness priming in men because of the same-sex experimental sessions (chosen in order to exclude the confounding effect of more or less attractive opposite-sex co-players), and in short, there were no “real” members of the opposite sex to impress with the generosity of an individual. However, running mixed-sex sessions could introduce stochastic noise (and potentially even systemic bias if predominantly less/more attractive women or men attended) into the results, and viewing photographs of members of the opposite sex directly before playing the experimental games should suffice to elicit an unconscious response to the presence of opposite sex (akin to the effect of “watching eyes” cues, reported by Haley and Fessler, 2005 and others). The immediacy of the games following the priming should also increase the effect, since long-exposure cues can cause habitation and weaker effects (Sparks and Barclay, 2013). A careful comparison of the effect of real-life (un)attractive observers and comparably (un)attractive images is needed in the future and constitutes an interesting line of research studies, akin to discerning nuances in the effects of observer cues brought up above (such as printed eyes) and real-life observers.

Allocations of DG in this study correlated positively with agreeableness (in line with Ben-Ner et al., 2004), and UG offers in the full sample were predicted by extraversion and neuroticism in interaction with sex, not unprecedented in previous literature (Ashton et al., 1998; Oda et al., 2014; Nakavachara, 2018), but these facts bear little relevance for costly signaling. However, the number of sexual partners in life was correlated to the MAOs in the UG: the higher the number, the higher was the MAO, as shown in Figure 2. A preference for an equal, or at least close-to-equal, split can indicate fairness (or inequity aversion), one of the psychological mechanisms of altruism, where rejecting an unfair offer acts as costly punishment. However, it is not necessarily altruistic, as it could also serve as a mechanism for avoiding the imposition of inferior status, rather than a prosocial act of punishing the responders so that they would behave more equitably in future interactions. While a cross-cultural study by Henrich et al. (2006) found a correlation between rejection rates in the UG and altruistic behavior in the DG, several other studies failed to find a link between costly punishment in the UG and prosocial behavior in other experimental games and situations (Ohmura and Yamagishi, 2005; Yamagishi et al., 2012; Brethel-Haurwitz et al., 2016) or failed to find meaningful third-party punishment, which could be considered altruistic (Pedersen et al., 2013) from an evolutionary, if not psychological, point of view.

These findings are in line with the current study, since sexual selection should lead to avoidance of being ascribed inferior social status. A correlation between DG allocation and MAO or between rejection of an unfair offer and MAO was not found, suggesting that UG rejections were more due to reputation-seeking rather than altruistic punishment. It is also in accordance with the positive relationship of self-perceived attractiveness and MAO in our sample. However, it could also be suggested that another mediating variable (such as self-esteem, which would likely be higher on average in people, especially men, with a greater number of sexual partners) was responsible for modulating the UG rejections and MAO. Self-esteem had been previously linked to altruistic behavior (but not necessarily altruistic motivations, see Batson et al., 1986; on the other hand, studies in different contexts did not find a common pattern, and the question of the hen and egg—whether self-esteem precedes altruistic behavior or the other way around, or the relationship of these traits goes in both directions—is not entirely clear; see e.g., Hessing and Elffers, 1985; Jiang et al., 2017).

In conclusion, we did not find evidence of attractiveness cues increasing altruistic behavior as predicted by the costly signaling theory. Women who had participated in this study behaved in the opposite direction; in men, the effect theoretically ran in the expected direction but was not significant. However, MAOs of UG correlated with the number of self-reported sexual partners, which is in line with the costly signaling theory. The findings highlight the need for replication of “well-known” effects (Ioannidis, 2005; Loken and Gelman, 2017). Further research studies are needed to address the relationships between the attractiveness of sexual cues with altruistic behavior and to shed more light on the role of sexual selection in the evolution of human altruism.

## Data Availability Statement

The datasets presented in this study can be found in online repositories. The names of the repository/repositories and accession number(s) can be found at: FigShare: doi.org/10.6084/m9.figshare.12067407.

## Ethics Statement

The study was approved by the research ethics board of the Faculty of Science, Charles University. The study was performed in accordance with the ethical standards as laid down in the 1964 Declaration of Helsinki and its later amendments. The patients/participants provided their written informed consent to participate in this study.

## Author Contributions

JN: study conception, participant invitation, data collection, analysis, and writing the paper. KM, KS, and VZ: data collection and comments on the analysis and paper. JF: study conception and assistance with analysis and writing the paper. All authors contributed to the article and approved the submitted version.

## Conflict of Interest

The authors declare that the research was conducted in the absence of any commercial or financial relationships that could be construed as a potential conflict of interest.
