# Supplementary material for: Looking Like a Million Dollars: Does Attractiveness Priming Increase Altruistic Behavior in Experimental Games?
Source: Front Psychol. 2021 Jul 20;12:658466. doi: 10.3389/fpsyg.2021.658466 (PMC8335640; doi:10.3389/fpsyg.2021.658466)
Supplement: Supplementary file 1 [file Table_1.docx]

Looking like a million dollars:

Does attractiveness priming increase altruism in experimental games?

Julie Novakova^1*^, Kamila Machová^1^, Kateřina Sýkorová^1^, Vojtěch Zíka^1,2^, Jaroslav Flegr^1,3^

^1^ Laboratory of Evolutionary Biology, Department of Philosophy and History of Science, Faculty of Science, Charles University, Prague, Czech Republic

^2^ Center for Behavioral Experiments (CEBEX)

^3^ National Institute of Mental Health, Klecany, Czech Republic

^*^ Corresponding author

Julie Novakova

[julie.novakova@natur.cuni.cz](mailto:julie.novakova@natur.cuni.cz)

**Supplementary Material**

Table S1. Differences of facial images from the priming sample in other descriptions by attractiveness.

|  | Male faces rated by women | | | | | | Female faces rated by men | | | | |
| --- | --- | --- | --- | --- | --- | --- | --- | --- | --- | --- | --- |
| Rated trait/description | Mean (unattractive group) | Mean (attractive group) | t value  (one-sided) | df | p value  (one-sided) | Mean (unattractive group) | | Mean (attractive group) | t value  (one-sided) | df | p value  (one-sided) |
| nice | 3.323039 | 4.349524 | -4.4149 | 37.723 | 4.097e-05 | 3.535831 | | 5.270525 | 9.0976 | 37.367 | 2.582e-11 |
| ideal partner for a one-night stand | 1.527654 | 2.371788 | -5.6869 | 28.176 | 2.086e-06 | 2.120 | | 4.639 | -10.204 | 27.512 | 3.734e-11 |
| ideal life partner | 1.846173 | 2.956122 | -6.0817 | 25.082 | 1.159e-06 | 2.257071 | | 4.780303 | -8.3783 | 28.354 | 1.855e-09 |
| sexy | 2.698418 | 3.968987 | -7.1816 | 35.313 | 1.06e-08 | 2.800000 | | 5.246923 | -9.6199 | 33.106 | 2.049e-11 |
| charismatic | 2.600225 | 3.835135 | -5.5567 | 30.909 | 2.197e-06 | 2.8515 | | 4.7495 | -8.4038 | 36.231 | 2.475e-10 |
| intelligence | 3.775798 | 4.653989 | -3.6664 | 37.939 | 0.000375 | 3.891241 | | 4.625182 | -4.5419 | 35.659 | 3.069e-05 |
| fun | 3.098451 | 3.923230 | -4.3287 | 36.934 | 5.494e-05 | 3.155895 | | 4.521834 | -7.4627 | 36.707 | 3.632e-09 |
| rich | 3.713853 | 4.517532 | -3.55 | 37.845 | 0.0005247 | 3.696332 | | 4.658301 | -3.9142 | 37.659 | 0.0001838 |
| generous | 3.958065 | 4.510215 | -2.4628 | 36.471 | 0.009318 | 4.099793 | | 4.830083 | -4.1058 | 35.107 | 0.0001143 |
| altruistic | 3.847030 | 4.417079 | -2.244 | 36.255 | 0.01551 | 4.094084 | | 4.778053 | -3.479 | 37.967 | 0.0006398 |
| nice | 3.971827 | 4.555838 | -2.6288 | 36.125 | 0.006252 | 4.108484 | | 5.067148 | -5.6893 | 35.11 | 9.813e-07 |
| trustworthy | 3.554808 | 4.381090 | -3.4591 | 37.813 | 0.0006789 | 3.756154 | | 4.921538 | -5.8674 | 33.285 | 6.908e-07 |
| healthy | 4.756774 | 5.933226 | -5.4134 | 31.315 | 3.182e-06 | 4.116154 | | 5.675385 | -6.8696 | 33.555 | 3.505e-08 |
| good skin quality | 4.241045 | 4.968657 | -2.6391 | 33.666 | 0.00625 | 4.001786 | | 5.046429 | -3.6596 | 37.993 | 0.0003821 |
| symmetry | 4.425000 | 4.863571 | -1.9996 | 36.715 | 0.02649 | 4.370968 | | 5.293548 | -5.4277 | 35.756 | 2.063e-06 |
| faithful | 4.688942 | 4.609856 | 0.35138 | 37.96 | 0.6364 | 4.797826 | | 4.636739 | 0.89705 | 37.625 | 0.1877 |
| mature | 5.130446 | 5.040099 | 0.20935 | 37.834 | 0.4176 | 5.607692 | | 4.182943 | 4.8231 | 37.855 | 1.161e-05 |
| masculine | 5.491885 | 5.358639 | 0.49285 | 33.7 | 0.3127 | 4.484967 | | 3.221405 | 5.4896 | 37.947 | 1.432e-06 |
| dominant | 4.661559 | 4.671237 | -0.03464 | 37.895 | 0.5137 | 4.382542 | | 4.200000 | 0.84037 | 34.937 | 0.2032 |

The table shows t-test comparisons of the attractive and unattractive priming sample images of men and women, respectively, in other characteristics. Each was rated by several hundred subjects in a study by Machová (2018). The two samples differ significantly in the majority of traits. However, they exhibited no substantial difference in dominance and only women showed a significant difference in maturity between the samples, making the dominance or age explanations for the negative effect of attractiveness priming unlikely.
